# Reviewers

**DOI:** 10.1111/cas.15670

**Published:** 2022-12-13

The publication of invaluable papers in Cancer Science depends on the prompt, careful review of submitted manuscripts. We would like to thank the following experts for reviewing manuscripts submitted between October 1, 2021 to September 30, 2022.

Reviewer names

Hiroyuki Abe

Tatsuya Abe

Jun Adachi

Keishi Adachi

Dennis Adeegbe

Antoine Adenis

Hiroshi Aikata

Shinichi Aishima

Kiwamu Akagi

Shusuke Akamatsu

Shinya Akatsuka

Yoshiki Akatsuka

Jun Akiba

Kazunori Akimoto

Masashi Akiyama

Yoshimitu Akiyama

Masashi Ando

Mizuo Ando

Kenjiro Aogi

Takashi Aoi

Koji Aoki

Naoko Aragane

J. B. Aragon‐Ching

Tomio Arai

Yoshiki Arakawa

Marito Araki

Hany Ariffin

Takaaki Arigami

Ken Asada

Noboru Asada

Akira Asai

Yoshinari Asaoka

Miyuki Azuma

Eishi Baba

Hideo Baba

Yoshifumi Baba

Kouji Banno

Lyudmila Bel'Skaya

Fabrizio Bianchi

Amina Bolatkan

Josep Borras

Zhixiong Cai

Rafael De La Camara

Ylenia Capodanno

Carlo Catapano

Rui‐Chao Chai

Sheng‐Chieh Chan

Yanzhong Chang

Chien‐Wen Chen

Fengsheng Chen

Guo Chen

Jen‐Shi Chen

Jianfeng Chen

Kai Chen

Ku‐Chung Chen

Ying Chen

Yongbin Chen

Yun Chen

Hailing Cheng

Quan Cheng

Natsuko Chiba

Kazuaki Chikamatsu

Shunsuke Chikuma

Go‐Eun Choi

Yoshiro Chuman

Amedeo Columbano

Shingo Dan

Markus Diener

Cinzia Domenicotti

Takahiro Domoto

Guangshi Du

Wushuang Du

Takashi Ebihara

Hidetaka Eguchi

Hidetoshi Eguchi

Takanori Eguchi

Shogo Ehata

Takeshi Emura

Makoto Endo

Daisuke Ennishi

Hideki Enokida

Tomohiro Enokida

Keisuke Enomoto

Masato Enomoto

Taito Esaki

Heng‐Yu Fan

Weiyi Fang

Yibin Feng

Isacco Ferrarini

Jorge Filmus

Caroline Ford

Shigeo Fuji

Satoshi Fujii

Shin‐Ichiro Fujii

Yoichi Fujii

Akihiro Fujimoto

Ayumi Fujimoto

Daichi Fujimoto

Kiyohide Fujimoto

Naohiro Fujimoto

Nobukazu Fujimoto

Mitsuhiro Fujishiro

Teruaki Fujishita

Kazutoshi Fujita

Kohei Fujita

Yu Fujita

Hiroshi Fujiwara

Naoto Fujiwara

Yoshiyuki Fujiwara

Yukio Fujiwara

Yutaka Fujiwara

So‐Ichiro Fukada

Katsumi Fukamachi

Akihisa Fukuda

Shinji Fukuda

Takeshi Fukuda

Hiroshi Fukuhara

Suguru Fukuhara

Noriyoshi Fukushima

Satoshi Fukushima

Takuya Fukushima

Wakaba Fukushima

Itoh Fumiko

Tatsuhiko Furukawa

Toru Furukawa

Nozomu Fuse

Manabu Futamura

Yuanhong Gao

Zuhua Gao

François Ghiringhelli

Min Gi

Akiteru Goto

Atsushi Goto

Hidemasa Goto

Jianguo Gu

Peng Guo

Tsuyoshi Hachiya

Junzo Hamanishi

Ichiro Hanamura

Hiroshi Handa

Megumi Hara

Yasuaki Harabuchi

Hiroshi Harada

Mamoru Harada

Toshiyuki Harada

Kenichi Harano

Koji Haratani

Naoya Hashimoto

Yutaka Hata

Shigetsugu Hatakeyama

Shingo Hatakeyama

Etsuro Hatano

Koji Hatano

Naoko Hattori

Fumihiko Hayakawa

Yoku Hayakawa

Hidetoshi Hayashi

Hideyuki Hayashi

Takuo Hayashi

Yoshihiro Hayashi

Johannes Haybäck

Shoichi Hazama

Hui‐Ying He

Yasuhiro Hida

Naoki Hijiya

Hayato Hikita

Masayuki Hino

Shinjiro Hino

Kunihiko Hinohara

Takao Hinoi

Toru Hiraga

Hideyo Hirai

Yusuke Hiraku

Kosuke Hiramatsu

Masaki Hirano

Nobuyoshi Hiraoka

Akira Hirasawa

Eishu Hirata

Hidenari Hirata

Kenji Hirata

Makoto Hirata

Akiyoshi Hirayama

Yoshihiko Hirohashi

Shuichi Hironaka

Yuichi Hirose

Kiichi Hirota

Yuichi Hisamatsu

Shigeo Hisamori

Takaki Hiwasa

Hiroaki Honda

Masao Honda

Yuto Honda

Fumiya Hongo

Tsunaki Hongu

Satoshi Honjo

Naoko Honma

Kuniko Horie

Masafumi Horie

Keisuke Horiuchi

Naoki Hosen

Takayuki Hoshii

Ayuko Hoshino

Daisuke Hoshino

Makoto Hosono

Satoyo Hosono

Yasuyuki Hosono

Noriko Hosoya

Md Amir Hossain

Katsuyuki Hotta

Che Yuan Hu

Dongsheng Hu

Qingjiang Hu

Rong Hu

Bo Huang

Gang Huang

Zhaohui Huang

Daisuke Ichikawa

Yoshinobu Ichimura

Masashi Idogawa

Kazuhiko Igarashi

Tsukasa Igawa

Yoshito Ihara

Keiichiro Iida

Hidekazu Iioka

Hisashi Iizasa

Hideaki Ijichi

Jun‐Ichiro Ikeda

Sadakatsu Ikeda

Satoshi Ikeda

Toru Ikegami

Yuzuru Ikehara

Tsuneo Ikenoue

Masashi Ikutani

Shinya Imada

Chihaya Imai

Hiroo Imai

Hisao Imai

Toshio Imai

Yoichi Imai

Toshihiko Imamura

Tatsuhiko Imaoka

Teruo Inamoto

Daichi Inoue

Jun Inoue

Keiji Inoue

Yasumichi Inoue

Takashi Inozume

Noriyoshi Iriyama

Fumihiro Ishida

Tatsuhiro Ishida

Jun Ishihara

Hiroki Ishii

Ken J Ishii

Kenichiro Ishii

Eiichi Ishikawa

Hitoshi Ishikawa

Mitsuya Ishikawa

Yuichi Ishikawa

Takatsugu Ishimoto

Akira Ishisaki

Toru Ishitani

Kenji Ishitsuka

Kenichi Ishizawa

Takeshi Isobe

Kota Itahashi

Hiroaki Itamochi

Akihiro Ito

Fumiya Ito

Hidemi Ito

Ken‐Ichi Ito

Kosei Ito

Shigeki Ito

Takaaki Ito

Takahiro Ito

Takeshi Ito

Yuri Ito

Fumiko Itoh

Shinji Itoh

Kazuya Iwabuchi

Atsushi Iwama

Eiji Iwama

Hiroto Izumi

Kouji Izumi

Masashi Izumiya

Koji Izutsu

Anand Jeyasekharan

Honglin Jin

Masatoshi Jinnin

Minji Jo

Yoshikazu Johmura

Yasuaki Kabe

Norimitsu Kadowaki

Shigenori Kadowaki

Hiroshi Kagamu

Hidenori Kage

Yuki Kagoya

Tsuneyasu Kaisho

Taisuke Kajino

Hiroaki Kajiyama

Yoshihiro Kakeji

Nobuyuki Kakiuchi

Yoshihiro Kamada

Yoichiro Kamatani

Yasuhiko Kamikubo

Masashi Kanai

Yoshikatsu Kanai

Takayuki Kanaseki

Hiroaki Kanda

Shintaro Kanda

Teru Kanda

Shin Kaneko

Shuzo Kaneko

Dongchon Kang

Gyeong Hoon Kang

Mitsunobu Kano

Shin‐Ichi Kano

Masato Karayama

Yoshinobu Kariya

Kennosuke Karube

Katsumi Kasashima

Ryoko Katagiri

Hiroaki Kataoka

Naoyuki Kataoka

Kazuhiro Katayama

Junya Kato

Kikuya Kato

Mamoru Kato

Motohiro Kato

Shingo Kato

Takuya Kato

Yukinari Kato

Hiroto Katoh

Keisuke Katsushima

Hiroo Katsuya

Manabu Kawada

Masataka Kawai

Taketo Kawai

Hisato Kawakami

Shigeru Kawakami

Yoshihiro Kawasaki

Atsunari Kawashima

Masahiro Kawashima

Daisuke Kawauchi

Masahito Kawazu

Hino Keisuke

Hirotsugu Kenmotsu

Hiroyasu Kidoya

Hirotoshi Kikuchi

Yoshinao Kikuchi

Yoshikane Kikushige

Takahiro Kimura

Keita Kirito

Hiroyuki Kishi

Toshihiro Kishikawa

Masatoshi Kitagawa

Hiroshi Kitajima

Shojiro Kitajima

Shunsuke Kitajima

Hidemitsu Kitamura

Hiroshi Kitamura

Masayuki Kitano

Jiro Kitaura

Joji Kitayama

Katsuyuki Kiura

Etsuko Kiyokawa

Yuko Kiyosue

Naomi Kiyota

Kazuma Kiyotani

Akira Kobayashi

Hiroya Kobayashi

Hisataka Kobayashi

Miho Kobayashi

Motohiro Kobayashi

Satoshi Kobayashi

Susumu Kobayashi

Takashi Kobayashi

Toshiyuki Kobayashi

Takahiro Kodama

Yoshinori Kodama

Yuzo Kodama

Hironori Koga

Tomoyuki Koga

Takahiro Kogawa

Yasuhiro Koh

Kenichi Kohashi

Susumu Kohno

Shinji Kohsaka

Yusuke Koizumi

Yu‐Ichiro Koma

Masaaki Komatsu

Shuhei Komatsu

Yoshito Komatsu

Shinichi Komiyama

Daisuke Komura

Kazumasa Komura

Eisei Kondo

Satoru Kondo

Akimitsu Konishi

Hideyuki Konishi

Masamitsu Konno

Koji Kono

Chihaya Koriyama

Naohiko Koshikawa

Ai Kotani

Daisuke Kotani

Masashi Koto

Yuta Kouyama

Junji Koya

Shohei Koyama

Takafumi Koyama

Yuriko Koyanagi

Toshiyuki Kozuki

Nina‐Naomi Kreis

Terufumi Kubo

Yasushi Kubota

Takahiro Kuchimaru

Yasusei Kudo

Yotaro Kudo

Kensuke Kumamoto

Tsutomu Kume

Kei Kunimasa

Hirofumi Kuno

Chihhsi Kuo

Yutaka Kurebayashi

Kiyonori Kuriki

Sei Kuriyama

Junya Kuroda

Masahiko Kuroda

Fumio Kurosaki

Daisuke Kurotaki

Kazuhiko Kurozumi

Masahiko Kusumoto

Shigeru Kusumoto

Takeshi Kuwata

George Lambrou

Jae‐Hoon Lee

Chi Yan Leung

Chia‐Yang Li

Gang Li

Jian‐Ming Li

Jing Li

Ke Li

Lianbo Li

Xuri Li

Huichun Liang

Peng Liao

Qianjin Liao

Yiyun Lin

Hong Liu

Suling Liu

Weiguang Liu

Zeyi Liu

Siew‐Kee Low

Jun Lu

Xiao‐Jie Lu

Shiwen Luo

Junyan Ma

Mitsuhiro Machitani

Daichi Maeda

Shin Maeda

Tomohiko Maehama

Takeo Maekawa

Makoto Maemondo

Nako Maishi

Hideki Makinoshima

Hideki Makishima

Shinichi Makita

Atsushi Manabe

Yoshimasa Maniwa

Jacopo Mariotti

Yoshiaki Maru

Dai Maruyama

Reo Maruyama

Tetsuo Mashima

Muneyuki Masuda

Shinobu Masuda

Takaaki Masuda

Yohei Masugi

Kenta Masui

Toshihiko Masui

Takashi Masuko

Mitsuko Masutani

Daisuke Matsubara

Eri Matsubara

Jun Matsubayashi

Noriyuki Matsuda

Yoko Matsuda

Kazuhiro Matsumoto

Koji Matsumoto

Kunio Matsumoto

Shinji Matsumoto

Yoshihisa Matsumoto

Noriomi Matsumura

Yukinori Matsuo

Hirokazu Matsushita

Yosuke Matsushita

Juntaro Matsuzaki

Atsushi Matsuzawa

Delia Mezzanzanica

Yi Miao

Yutaka Midorikawa

Shuji Mikami

Daiki Miki

Hiroaki Miki

Kosuke Mima

Yosuke Minami

Yoji Minamishima

Toshinari Minamoto

Mari Mino‐Kenudson

Aya Misawa

Saori Mishima

Yuichi Mitobe

Yoichiro Mitsuishi

Ayako Mitsuma

Shuichi Mitsunaga

Masahiko Miura

Satoru Miura

Etsuko Miyagi

Yohei Miyagi

Makito Miyake

Shohei Miyamoto

Shigeo Miyata

Eisaku Miyauchi

Shiro Miyayama

Akihiro Miyazaki

Hiroaki Miyoshi

Tomoichiro Miyoshi

Yusuke Mizukami

Masao Mizuki

Eishiro Mizukoshi

Masamichi Mizuma

Ryuichi Mizuno

Tdahaya Mizuno

Tsunekazu Mizushima

Isao Momose

Shuji Momose

Yukihide Momozawa

Seiichi Mori

Masato Morikawa

Masahiro Morise

Satoko Morishima

Kazuhiro Morishita

Yoshifumi Morita

Chigusa Morizane

Toshiro Moroishi

Shinichiro Motohashi

Ken‐Ichi Mukaisho

Toru Mukohara

Junko Mukohyama

Junko Murai

Kazunori Murai

Takashi Murakami

Yoshiki Murakami

Yoshiki Murakumo

Daisuke Muraoka

Yoji Murata

Masafumi Muratani

Taisei Mushiroda

Manabu Muto

Michihiro Mutoh

Shigeyuki Nada

Genta Nagae

Shigenori Nagai

Hiroaki Nagano

Osamu Nagano

Koji Nagaoka

Kazunori Nagasaka

Satoru Nagase

Kengo Nagashima

Satoshi Nagayama

Isao Naguro

Hisamichi Naito

Yuji Naito

Yutaka Naito

Hayato Nakagawa

Masahiro Nakagawa

Masao Nakagawa

Satoshi Nakagawa

Takashi Nakagawa

Takuya Nakagawa

Kenji Nakahama

Yoshiro Nakahara

Shingo Nakahata

Yousuke Nakai

Masao Nakajima

Yasunari Nakamoto

Kenichi Nakamura

Naoya Nakamura

Seigo Nakamura

Takafumi Nakamura

Takanori Nakamura

Takashi Nakamura

Yoshiaki Nakamura

Yuki Nakanishi

Hiroyasu Nakano

Yusuke Nakano

Takashi Nakaoku

Chiaki Nakaseko

Masahiro Nakashima

Yoko Nakasu

Fumihiko Nakatani

Munehide Nakatsugawa

Hirokazu Nakatsumi

Keiko Nakayama

Masafumi Nakayama

Masashi Nakayama

Mizuho Nakayama

Takeo Nakayama

Takeshi Namekawa

Takeshi Namiki

Asuka Nanbo

Yasuhito Nannya

Shintaro Narita

Manabu Natsumeda

Naohiko Nazai

Lui Ng

Atsushi Niida

Naoki Niikura

Keisuke Nimura

Jun Nishida

Mikako Nishida

Naohiro Nishida

Jun Nishikawa

Tadaaki Nishikawa

Yuji Nishikawa

Momoko Nishikori

Meiko Nishimura

Tatsunori Nishimura

Hiroshi Nishina

Takashi Nishina

Tomohiro Nishina

Hitomi Nishinakamura

Yoshikazu Nishino

Makoto Nishio

Nobuhiro Nishio

Shin Nishio

Yasuhiko Nishioka

Michiru Nishita

Hideki Nishitoh

Noyuki Nishiya

Kohji Noguchi

Takuro Noguchi

Hiroshi Nokihara

Takahiro Nomoto

Sachiyo Nomura

Rintaro Noro

Kisato Nosaka

Kaname Nosaki

Hideaki Obara

Wataru Obara

Daisuke Obinata

Sadahisa Ogasawara

Kumiko Ogawa

Mikako Ogawa

Hisakazu Ogita

Takashi Ohama

Yuuki Ohara

Kadoaki Ohashi

Akihiro Ohba

Shigeo Ohba

Tomokazu Ohishi

Fumiharu Ohka

Takayuki Ohkuri

Tohru Ohmori

Shinobu Ohnuma

Nobumichi Ohoka

Shouzou Ohsumi

Shigeki Ohta

Tomohiko Ohta

Masayuki Ohtsuka

Takao Ohtsuka

Sumio Ohtsuki

Kenoki Ohuchida

Tsukasa Oikawa

Yuichi Oike

Satoshi Oizumi

Yusuke Oji

Nobuyuki Okahashi

Aikou Okamoto

Susumu Okano

Toshihiko Okazaki

Yasumasa Okazaki

Eiji Oki

Yukari Okita

Shujiro Okuda

Yoshinaga Okugawa

Yusuke Okuma

Chitose Oneyama

Rikiya Onimaru

Nobuyuki Onishi

Makiko Ono

Mayumi Ono

Shunsuke Ono

Makiko Ono‐Fuchiwaki

Kunishige Onuma

Akira Orimo

Mitsuhiko Osaki

Shigehito Osawa

Tsuyoshi Osawa

Hiroko Oshima

Shigeru Oshima

Atsushi Osoegawa

Franz Oswald

Motoyuki Otsuka

Naohide Oue

Noriko Ouji‐Sageshima

Yuko Oya

Jun Oyanagi

Shuji Ozaki

Toshinori Ozaki

Hiroaki Ozasa

Isao Oze

Pratheeba Palasuberniam

Cheng Pan

Jae‐Hyun Park

Jeffrey Parvin

Abhijit Patra

Andrea Piccin

Lucas Pontel

Xiaojian Qiu

Ramandeep Rattan

Masaki Ri

Christine Robin

Susumu Rokudai

Aileen G. Rowan

Hisataka Sabe

Mahito Sadaie

Hiroshi Saeki

Ken Saijo

Kumiko Saika

Akira Saito

Motonobu Saito

Rintaro Saito

Toshiyuki Saito

Yoichi Saito

Yoshimasa Saito

Masao Saitoh

Masakiyo Sakaguchi

Akira Sakai

Juro Sakai

Katsuya Sakai

Kazuko Sakai

Tetsuya Sakai

Akio Sakamoto

Naoya Sakamoto

Shinichi Sakamoto

Takeharu Sakamoto

Shingo Sakashita

Tomohisa Sakaue

Hideyuki Sakurai

Hiroaki Sakurai

Arturo Sala

Oltea Sampetrean

Masashi Sanada

Takaomi Sanda

Daisuke Sano

Tetsuro Sasada

Toshiyuki Sasagawa

Hiroki Sasaki

Koji Sasaki

Nobunari Sasaki

So‐Ichiro Sasaki

Takaaki Sasaki

Takashi Sasayama

Eiichi Sato

Mitsuo Sato

Shinya Sato

Yasuharu Sato

Yusuke Sato

Taroh Satoh

Masahiro Seike

Motoaki Seki

Naohiko Seki

Yoshitaka Seki

Ikuo Sekine

Keisuke Sekine

Shigeki Sekine

Yusuke Sekine

Heloisa Selistre‐De‐Araujo

Bao‐En Shan

Yongping Shao

Tang‐Long Shen

Aya Shiba

Kiyotaka Shiba

Atsushi Shibata

Makoto Shibutani

Akira Shibuya

Kotaro Shide

Tadahiko Shien

Kiyoto Shiga

Takashi Shiina

Kazuyuki Shimada

Midori Shimada

Shu Shimada

Yasuhiro Shimada

Yoshihisa Shimada

Hiroaki Shime

Katsuhiko Shimizu

Toshio Shimizu

Masayuki Shimoda

Tatsunori Shimoi

Tomoya Shimokata

Akihiko Shimomura

Osamu Shimozato

Takero Shindo

Kato Shinichiro

Keiko Shinjo

Naoki Shinojima

Yasushi Shintani

Daisuke Shiokawa

Takayuki Shiomi

Masaki Shiota

Atsushi Shiozaki

Tanri Shiozawa

Kouya Shiraishi

Toshiro Shirakawa

Masayuki Shirasawa

Hidekazu Shirota

Kohei Shitara

Masayuki Sho

Yongqian Shu

Eric Snyder

Kenzo Soejima

Yuji Soejima

Junichi Soh

Lilja B. Solnes

Yukihiko Sonoda

Takashi Sonoki

Masahiro Sonoshita

Yoshihiro Sowa

Kenichi Suda

Yasuo Suda

Kazuki Sudo

Tomoya Sudo

Yasuhito Suehara

So‐Ichi Suenobu

Tamotsu Sugai

Kokichi Sugano

Keishi Sugimachi

Akira Sugimoto

Mikio Sugimoto

Daisuke Sugiura

Kazuhiko Sugiyama

Kentaro Suina

Hidetoshi Sumimoto

Jiying Sun

Zhi‐Jun Sun

Yu Sunakawa

Kuniko Sunami

Ayako Suzuki

Hidefumi Suzuki

Hiromichi Suzuki

Hiroshi Suzuki

Hiroyuki Suzuki

Keiji Suzuki

Miho Suzuki

Mikiko Suzuki

Norio Suzuki

Ryo Suzuki

Sadao Suzuki

Shigeaki Suzuki

Shugo Suzuki

Takafumi Suzuki

Takeshi Suzuki

Yoshiyuki Suzuki

Sho Tabata

Takashi Taga

Hiroyuki Tagawa

Ayumi Taguchi

Ayumu Taguchi

Keiko Taguchi

Satoru Taguchi

Naruto Taira

Ken Tajima

Masahiro Takada

Masatoshi Takagi

Satoshi Takagi

Takayuki Takahama

Akihisa Takahashi

Akiko Takahashi

Hirotaka Takahashi

Kei Takahashi

Masanobu Takahashi

Nobuaki Takahashi

Ryou‐U Takahashi

Satoshi Takahashi

Shin Takahashi

Takeo Takahashi

Toshiaki Takahashi

Tsuyoshi Takahashi

Atsushi Takai

Manabu Takamatsu

Akiyoshi Takami

Hirokazu Takami

Shinichi Takano

Akinori Takaoka

Ken Takasawa

Hiroki Takashima

Atsushi Takatori

Kenichi Takayama

Koichi Takayama

Tetsuji Takayama

Shunsaku Takayanagi

Haruna Takeda

Kazuyoshi Takeda

Takuya Takeichi

Toshihiko Takeiwa

Akira Takemoto

Hideyuki Takeshima

Fumitaka Takeshita

Akinobu Taketomi

Masashi Takeuchi

Shinji Takeuchi

Yasuto Takeuchi

Yuichi Takiguchi

Takahisa Takino

Jun Takizawa

Keiyo Takubo

Satoshi Tamada

Keiichi Tamai

Kentaro Tamaki

Mayumi Tamari

Hideto Tamura

Kenji Tamura

Takaaki Tamura

Yasuaki Tamura

Fumihiro Tanaka

Keitaro Tanaka

Kentaro Tanaka

Masamitsu Tanaka

Masato Tanaka

Miwa Tanaka

Shinya Tanaka

Shota Tanaka

Takuji Tanaka

Toshiya Tanaka

Toshiyuki Tanaka

Yasuhito Tanaka

Faqin Tang

Shoichiro Tange

Tomohiko Taniai

Tetsuya Tanigawa

Hiroaki Taniguchi

Kohei Taniguchi

Koji Taniguchi

Toshiyasu Taniguchi

Chizu Tanikawa

Michihiro Tanikawa

Hirokazu Tanino

Kiyomi Taniyama

Junko Tanizaki

Yongguang Tao

Yusuke Tarumoto

Etsu Tashiro

Satoshi Tashiro

Keisuke Tateishi

Kensuke Tateishi

Ryosuke Tateishi

Shotaro Tatekawa

Hiroaki Tateno

Yasutoshi Tatsumi

Hiroshi Tazawa

Huajing Teng

Zhaogang Teng

Mitsuo Terada

Naoki Terada

Seitaro Terakura

Koji Teramoto

Yasuhito Terui

Kayo Togawa

Yuji Toiyama

Eriko Tokunaga

Fuminori Tokunaga

Akihiro Tomita

Arata Tomiyama

Takeo Toshima

Tatsuya Toyama

Hiroyoshi Tsubochi

Shoma Tsubota

Naoto Tsuchiya

Norihiko Tsuchiya

Masumi Tsuda

Akihito Tsuji

Takahiro Tsujikawa

Tomohide Tsukahara

Hirotake Tsukamoto

Tetsuya Tsukamoto

Kunihiro Tsukasaki

Tadasuke Tsukiyama

Junji Tsurutani

Takahiro Tsushima

Toyonori Tsuzuki

Tomoyuki Uchihara

Takeshi Uchiumi

Hibiki Udagawa

Keiko Udaka

Tetsuya Ueba

Yuri Ueda

Yutaka Ueda

Satoshi Ueha

Hisanori Uehara

Hiroji Uemura

Motohide Uemura

Masaya Ueno

Takayuki Ueno

Hisashi Uhara

Atsushi Umemura

Shigeki Umemura

Hiroshi Ureshino

Tetsuo Ushiku

Ryo Ushioda

Takeo Usui

Hisashi Wada

Keiko Wada

Satoshi Wada

Keiji Wakabayashi

Yuichi Wakabayashi

Tetsuro Wakasugi

Hiroaki Wakimoto

Anxun Wang

Min Wang

Ming‐Yu Wang

Wei Wang

Keisuke Watanabe

Nobumoto Watanabe

Satoshi Watanabe

Sugiko Watanabe

Takashi Watanabe

Yukihide Watanabe

Junaid Wazir

Seo Wooseok

Jianmin Wu

Chengying Xie

Hong Xie

Yuntao Xie

Shunbin Xiong

Ke Xu

Chenghao Xuan

Shigeki Yagyu

Tadaaki Yamada

Yasuhide Yamada

Wataru Yamagami

Hideki Yamaguchi

Hiroki Yamaguchi

Kazunori Yamaguchi

Ken Yamaguchi

Kiyoshi Yamaguchi

Motoko Yamaguchi

Kazuhito Yamamoto

Keisuke Yamamoto

Masahiro Yamamoto

Masato Yamamoto

Mizuki Yamamoto

Tomofumi Yamamoto

Yusuke Yamamoto

Ryuya Yamanaka

Shoji Yamaoka

Fumiyuki Yamasaki

Toshinari Yamasaki

Hiroko Yamashita

Keishi Yamashita

Masakatsu Yamashita

Satoshi Yamashita

Taro Yamashita

Tatsuya Yamashita

Yo‐Ichi Yamashita

Akira Yamauchi

Takahiro Yamauchi

Yoshio Yamauchi

Etsuko Yamazaki

Chunhong Yan

J Yan

Noriko Yanagitani

Hideyuki Yanai

Xiaoping Yang

Xiaoxu Yang

Shingo Yano

Tomonori Yano

Hiroyuki Yasuda

Jun Yasuda

Masahito Yasuda

Takahiko Yasuda

Takehiro Yasukawa

Jun‐Ichirou Yasunaga

Masahiro Yasunaga

Chi‐Tai Yeh

Kiyotaka Yoh

Takehiko Yokobori

Akira Yokoi

Sana Yokoi

Tomoyuki Yokose

Tadashi Yokosuka

Tomoya Yokota

Akihiko Yokoyama

Satoru Yokoyama

Takashi Yokoyama

Yasuhisa Yokoyama

Hiroshi Yokozaki

Kimio Yonesaka

Yasuto Yoneshima

Hiroshi Yoshida

Kenichi Yoshida

Kosuke Yoshida

Reiko Yoshida

Tatsuya Yoshida

Yoshinori Yoshida

Masato Yoshihara

Tetsuro Yoshimaru

Yasuhiro Yoshimatsu

Akihide Yoshimi

Koji Yoshimoto

Takayuki Yoshimoto

Yuya Yoshimoto

Akihiko Yoshimura

Kiyoshi Yoshimura

Tetsuhiro Yoshinami

Kiyoshi Yoshino

Kenichi Yoshioka

Yusuke Yoshioka

Hideyuki Yoshitomi

Hironori Yoshiyama

Tomoharu Yoshizumi

Junichiro Yuda

Mayu Yunokawa

Vesna Zadnik

Masayoshi Zaitsu

Mirjam B. Zeisel

Sadamoto Zenda

Yoshitaka Zenke

Cheng Zhan

Hong‐Liang Zhang

Hua Zhang

Jingjing Zhang

Jianda Zhou

